# Using Actiwatch to monitor circadian rhythm disturbance in Huntington’ disease: A cautionary note

**DOI:** 10.1016/j.jneumeth.2016.01.009

**Published:** 2016-05-30

**Authors:** Jenny Townhill, Alis C. Hughes, Benny Thomas, Monica E. Busse, Kathy Price, Stephen B. Dunnett, Michael H. Hastings, Anne E. Rosser

**Affiliations:** a The Cardiff University Brain Repair Group, Life Sciences Building, School of Biosciences, Museum Avenue, Cardiff CF10 3AX, United Kingdom; b Neuroscience and Mental Health Research Institute, Hadyn Ellis Building, Maindy Road, Cathays, Cardiff CF24 4HQ, United Kingdom; c Dept Neurolophysiology, University Hospital of Wales, Heath Park, Cardiff CF14 4XW, United Kingdom; d School of Healthcare Sciences, Cardiff University, 35-43 Newport Road, Cardiff CF24 OAB, United Kingdom; e MRC Laboratory of Molecular Biology, Cambridge Biomedical Campus, Francis Crick Avenue, Cambridge CB2 0QH, United Kingdom

**Keywords:** Actiwatch, Actimetry, EEG, Sleep circadian, Huntington's disease

## Abstract

Huntington's disease (HD) is an inherited neurodegenerative disorder that is well recognised as producing progressive deterioration of motor function, including dyskinetic movements, as well as deterioration of cognition and ability to carry out activities of daily living. However, individuals with HD commonly suffer from a wide range of additional symptoms, including weight loss and sleep disturbance, possibly due to disruption of circadian rhythmicity. Disrupted circadian rhythms have been reported in mice models of HD and in humans with HD. One way of assessing an individual's circadian rhythmicity in a community setting is to monitor their sleep/wake cycles, and a convenient method for recording periods of wakefulness and sleep is to use accelerometers to discriminate between varied activity levels (including sleep) during daily life. Here we used Actiwatch^®^ Activity monitors alongside ambulatory EEG and sleep diaries to record wake/sleep patterns in people with HD and normal volunteers. We report that periods of wakefulness during the night, as detected by activity monitors, agreed poorly with EEG recordings in HD subjects, and unsurprisingly sleep diary findings showed poor agreement with both EEG recordings and activity monitor derived sleep periods. One explanation for this is the occurrence of ‘break through’ involuntary movements during sleep in the HD patients, which are incorrectly assessed as wakeful periods by the activity monitor algorithms. Thus, care needs to be taken when using activity monitors to assess circadian activity in individuals with movement disorders.

## 1 Introduction

Huntington's disease (HD) is a progressive neurodegenerative disorder presenting in midlife with a triad of motor, emotional and cognitive symptoms (Walker, 2007). It is well established that the mutation causing HD affects the central nervous system, in particular, the medium spiny neurons of the striatum (Estrada Sánchez et al., 2008). The motor disorder is most widely known for the characteristic and striking involuntary movements, in particular chorea and dystonia, but the cognitive decline and emotional disturbance are often more debilitating. Moreover, there are a range of other symptoms such as weight loss and sleep disturbance that are commonly reported in HD and can be difficult to manage. Sleep disturbance can be distressing for both patients and carers, may exacerbate the cognitive decline, and is also reported in many other progressive neurodegenerative disorders such as Parkinson's disease (PD) and Alzheimer's disease (AD) (Aziz et al., 2010, Raggi et al., 2013, Slats et al., 2013). Sleep disturbance in HD has been reported as occurring very early in the course of the disease in both animal and human studies and is temporally associated with cognitive deterioration (Lazar et al., 2015, Lebreton et al., 2015). It is a disruptive symptom for both affected individuals and their carers and currently is by necessity treated empirically and often with little success (Morton, 2013). Although there is some evidence of degeneration of the hypothalamus in HD individuals (Van Wamelen et al., 2013, Hult et al., 2010) and evidence of lowered melatonin levels (Kalliolia et al., 2014) the mechanism underlying circadian rhythmicity disruption and sleep disturbance are still unclear and need to be elucidated in order to develop effective treatments (Morton, 2013).

There is evidence that both mouse and rat transgenic models of HD can recapitulate sleep disorders reported in patients, making them potentially important means for understanding the mechanisms underlying circadian disruption and sleep disturbance. For example, the circadian behaviour of R6/2 mice was found to be disturbed, with increased daytime and reduced nocturnal activity along with disruption of circadian clock genes (*mPer*2 and *mBmal1*) in the suprachiasmatic nuclei (SCN), motor cortex and striatum (Morton et al., 2005). A relationship between circadian disruption and cognitive decline in R6/2 HD mice was inferred when the pharmacological imposition of sleep and wakefulness with Alprazolam (Pallier et al., 2007) and Modafinil (Pallier and Morton, 2009) was shown to improve cognitive function in the mutant animals. Less work has been done in transgenic rat models, although a transgenic rat model of HD mirrors the sleep-wake disturbances seen in the mice, with accompanying reduction in the levels of adrenergic α2 receptors and leptin (Bode et al., 2009). The capacity to translate between human and rodent studies is important in facilitating understanding of the biology of circadian disruption in HD, and a number of techniques have been employed to investigate sleep architecture in HD patients and rodents, with polysonography being recognised as the gold standard with EEG as a necessary and important element of this. However, full polysonography, or even simple ambulatory EEG, are cumbersome, difficult to perform over prolonged periods of time for technical and acceptability reasons, and relatively expensive. Thus, there have been attempts use wearable devices such as Actiwatch^®^ to assess movement over one or more 24 h periods as a surrogate for sleep on the basis that individuals tend to move substantially more when they are awake than asleep. Indeed Actiwatch^®^ technology has been used in a number of HD circadian and sleep studies (Hurelbrink et al., 2005, Morton et al., 2005, Kantor et al., 2013) and could represent a simple and acceptable way of recording circadian rhythm and sleep in both people and animal models of HD. Here we compare Actiwatch^®^ recording, ambulatory EEG and sleep diaries in small numbers of HD individuals as a prelude to developing a translational platform to assess circadian disruption in this disorder. While the gold standard for sleep analysis is polysomnography (PSG), in our study we adapted and confined the test to EEG recording as we were interested in differentiating between wakefulness and sleep (rather than sleep scoring) this was considered sufficient and found to be pragmatic.

## 2 Materials and methods

### 2.1 Study population

Thirteen participants were recruited to this study from the South Wales Huntington's disease clinic, based in Cardiff. Inclusion criteria included a positive genetic test for HD, being above the age of 18 and below the age of 65, and having no concomitant medical conditions. Asymptomatic individuals were defined as having a Unified Huntington's Disease Rating Scale (UHDRS) total functional score (TFC) of 13/13 and an UHDRS motor score of less than 6 (*n* = 4), and symptomatic individuals having a TFC between 4 and 11 and a total motor score greater than 20 (*n* = 9). Assessment of disease status (UHDRS motor and functional scores) were undertaken by an experienced neurologist. Nine community control individuals were recruited. The demographics of the study participants are summarised in Table 1. In the presymptomatic group, one patient was on oral contraceptive and one on buproprion. Of the symptomatic patients, one was on an anti-psychotic, olanzapine, one on an antidepressant, two were on hypnotics, one on asprin and another on aspirin and ibroprofen. In the presymptomatic group there were 2 ex-smokers and 1 non-smokers. In the symptomatic group there were 2 ex-smokers, 1 smoker and 6 non-smokers. One symptomatic patient had a history of previous alcohol abuse and alcohol history was unremarkable for all other participants. No subjects had respiratory comorbidities.

Ethical approval for the study was obtained from the South East Wales local research ethics committee (08/WSE02/10) and patients were recruited from the Cardiff Huntington's disease clinic. All diagnoses were confirmed with a positive genetic test. Controls were healthy volunteers who were not at risk of HD. All individuals recruited into the study were asked to wear ambulatory EEGs for a 24 h period, to wear an Actiwatch^®^ and to keep a sleep diary for a period of one week, and to donate saliva samples for cortisol measurement.

The Actiwatch^®^ Activity monitoring system (Cambridge Neurotechnology Ltd) was worn on the non-dominant hand and recorded activity over a period of seven days. Actiwatch records with a sensitivity of 0.05 g with a bandwidth between 3 Hz and 11 Hz and a sampling frequency of 32 Hz. 1 min epochs were put into 5 min bins for comparison with EEG. The Actiwatches^®^ employed an analogue that used the amount of activity (number of movements per epoch, and number of movements above a predefined threshold) to make an estimate as to whether the subject was awake or asleep. All sleep episodes were visually inspected before analysis to screen for artefacts and malfunctioning. The watches were waterproof and participants were asked to wear them continuously. Patients were instructed to press the activation button, which delivered a single recorded pulse, to indicate the time they started to try to sleep and to press again to indicate waking in the morning.

EEG electrodes were fixed with collodion and placed according to the 10/20 international system. The continuous recording was obtained using ambulatory EEG (XLTEK) system worn for 24 h. The date and time were synchronised for analysis of EEGs and Actiwatch^®^ data. The EEG traces were analysed offline by an experienced neurophysiologist.

The EEG was scored manually in epochs of five minute to determine wakefulness or sleep. The criteria for wakefulness was denoted by presence of eyeblinks artefacts, and/or an alpha rhythm in the EEG. Early sleep stage was characterized by lack of eyeblink artefacts, fragmentation or absence of alpha activity and replacement of background EEG by low amplitude mixed frequency EEG activity and presence of slow eye movements. Later stages of sleep were identified by characteristic sleep phenomena such as vertex waves, K complexes, sleep spindles and slow waves. Differentiation between REM (rapid eye movement) sleep and wakefulness was determined by lack of eyeblink artefacts, absence of sustained alpha activity and presence of rapid eye movement artefacts.

Patients were given sleep diaries in the form of a booklet with a series of questions for each 24 h period. They were encouraged to make entries into the diary throughout the day with an emphasis on collecting information about night time sleep as soon as possible after rising in the morning. The questions included: time of going to bed at night; time the subject started trying to go to sleep; approximately how long it took them to fall asleep; how many times they woke in the night and the duration of the wakeful period; the time they woke in the morning; the time they rose in the morning; the number and duration of day time naps.

All participants collected saliva twice a day, 12 h apart (080.00 and 20.00), for a week by collecting saliva in microcentrifuge tubes. Subjects were requested to collect at least 1 ml of saliva (to a mark on the tube). Samples were stored at −20 °C until analysis of cortisol levels could be performed by Prof J Herbert and S Cleary (University of Cambridge).

## 3 Statistics

All ANOVA were undertaken using the Genstat v16.1 statistical package, with unbiased iterative correction for missing values. Comparison of epochs of Actiwatch^®^ wakefulness with EEG and sleep diaries was performed visually on a patient-by-patient basis.

## 4 Results

### 4.1 Sleep diaries

**Fig. 1:**
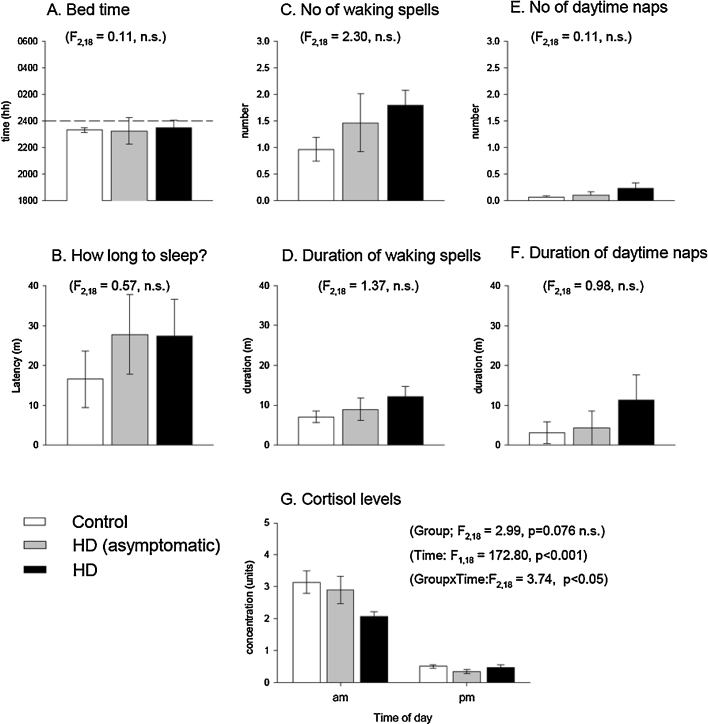
Sleep diary analysis. Analysis of sleep diary data in control, HD asymptomatic and HD symptomatic individuals. No statistically significant differences were found, although the trends may indicate a value for sleep diaries with larger group numbers. The error bars represent standard errors of the mean.

Of the 22 participants who took part in the study, 21 completed a sleep diary. There was a trend for deterioration across most parameters with more advanced disease state (Fig. 1A–F), in particular for sleep latency, number and duration of wakeful periods in the night, and number and duration of day time naps, but not for time of first attempting night-time sleep. However, when the groups were compared by 2-way ANOVA none of the differences reached significance on any of the parameters.

### 4.2 Actiwatch^®^ recordings

**Fig. 2:**
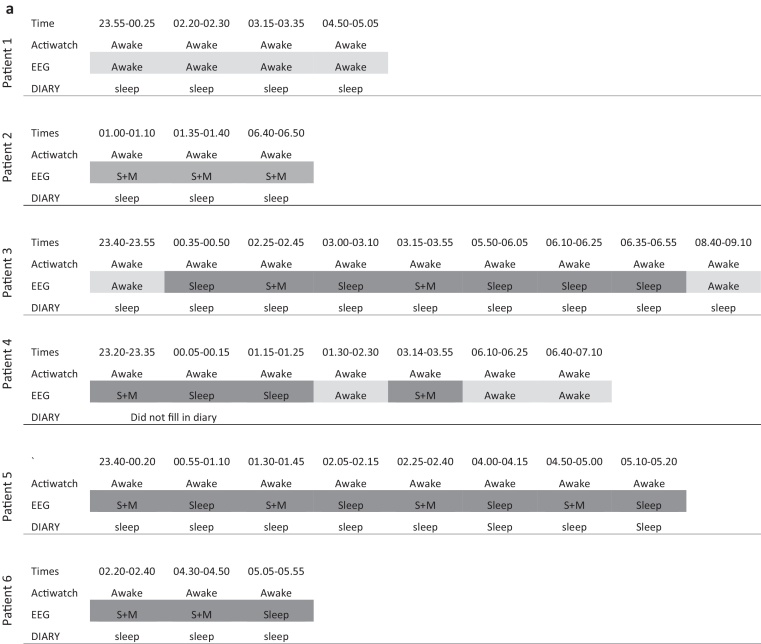

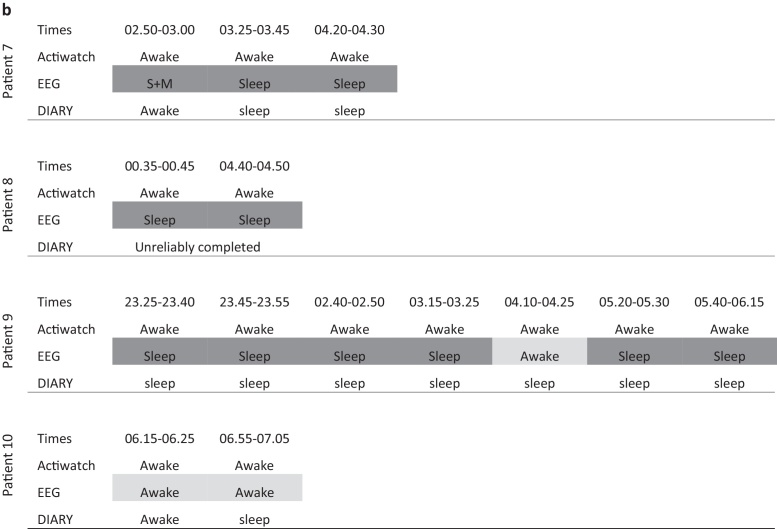
Periods of wakefulness as indicated by Actiwatch recordings in the six HD symptomatic individuals (a) and four asymptomatic individuals (b) in whom both recordings were available, along with at least one night recording in the sleep diary. No periods were seen in which the Actiwatch recorded sleep and the EEG recorded awake. Conversely, there were multiple periods when the Actiwatch recorded wakefulness that corresponded with “sleep” or “sleep plus movement artefact’ on the EEG (period of Actiwatch and EEG agreement in light grey and disagreement in dark grey). There is one period (patient 7) where the Actiwatch and sleep diary agree and the EEG recording has been interpreted as sleep plus movement. Abbreviations: S + M = sleep plus movement artefact.

There was some loss of data caused by water damage due to faulty waterproofing of some watches. Analysis of Actiwatch^®^ data by group (pre-symptomatic vs symptomatic vs controls) to assess differences in circadian rhythmicity did not reveal any significant group differences. The periods of wakefulness as assessed by Actiwatch^®^ were then directly compared to EEG and sleep diary data on a patient-by-patient basis (see Fig. 2).

### 4.3 Comparison of sleep diaries, Actiwatch^®^ and EEG recordings

As the EEG was fitted for the first 24 h of the 7 day experiment, the results obtained from the three different methods were compared over the first 24 h to assess the consistency between them in measuring sleep disturbance. For three of the symptomatic individuals both full Actiwatch^®^ and EEG data was not available and so they were not included in the analysis. Across all groups, the sleep diary had a tendency to fail to capture night-time waking as recorded by the EEG. All periods of sleep as determined by Actiwatch^®^ agreed with the EEG recording. However, for both symptomatic (Fig. 2) and asymptomatic (Fig. 3) HD subjects, periods of night-time wakefulness recorded by the Actiwatch^®^ agreed poorly with EEG recordings in that there were multiple periods of ‘wakefulness’ indicated by the Actiwatch^®^ for which the corresponding period of EEG recording demonstrated the individual to be asleep. This was not the case for all patients: in patient 1 (with 4 periods of waking) and patient 10 (with 2 periods of waking) there was agreement with the EEG recording. Some of these epochs were characterised by excessive movement artefact, and these are indicated in the figure as “sleep plus movement”. There was one period of wakefulness recorded by the Actiwatch^®^ that corresponded with a recording of awake in the sleep diary and sleep plus movement on the EEG (patient 7). This may indicate that the EEG recording of this epoch does not reliably indicate sleep, although this patient was noted to have completed the diary unreliably. Unfortunately a full data set was only available for one control patient (due to Actiwatch^®^ failure and shortage of EEG monitors for use in controls). In that subject there were two periods of Actiwatch^®^ waking; one associated with “sleep plus movement” on the EEG and one also recorded as wakefulness by the EEG.

### 4.4 Cortisol

Saliva samples were obtained for all 4 asymptomatic, 6 of the 9 symptomatic, and all nine control individuals. There were missing samples across the 7 days in many cases, and indeed only one of the controls produced a complete set of samples. Missing values were corrected by an iterative unbiased estimator routine within the analyses of variance package (Genstat v16.2, VSN International, Oxford). On direct questioning, many subjects of both the HD affected and control groups reported that they found saliva collection mildly unpleasant and also the required amount of saliva. Analysis of cortisol levels revealed no differences between groups (*F*_2,18_ = 2.99, *p* < 0.076), but a difference in time (*F*_1,16_ = 172.8, *p* < 0.001) with morning cortisol levels being higher than in the evening, and a significant group × time interaction (*F*_2_,_18_ _=_ 3.74, *p* < 0.05), with a blunted cortisol peak in HD symptomatic subjects (see Fig. 1G).

## 5 Discussion

By using three methods to monitor sleep in HD patients, this study highlights the potential shortcomings in diary and actimetry records, in comparison to the more intensive ambulatory EEG approach. The main finding in this study was that ambulatory EEG recordings suggested that caution should be applied when interpreting Actiwatch^®^ recording in individuals with both asymptomatic and symptomatic HD. Specifically, although there was good agreement when the Actiwatches^®^ indicated sleep, and there were periods in which both Actiwatches^®^ and EEG demonstrated the individual to be awake, there were also multiple occasions when the Actiwatch^®^ indicated wakefulness but the EEG indicated that the patients was asleep. This was the case even in those periods in which sleep recordings punctuated with movement artefact were excluded. Thus, in these cases recording with Actiwatches^®^ alone would overestimate the extent of wakefulness, even in asymptomatic subject with little day time chorea, which could consequently interfere with assessment of circadian rhythmicity. The most likely reason for this discrepancy is that, despite the fact that most involuntary movements in HD disappear with sleep, people with HD nevertheless display involuntary movements during lighter periods of sleep (Fish et al., 1991), and these are more exaggerated than sleep-associated movement in control individuals and are thus recorded as wakeful by the Actiwatch^®^ algorithm.

Thus, caution is necessary in interpreting Actiwatch^®^ assessment of wake/sleep cycles in individuals with movement disorders. It is possible that this could be addressed by adjustments to the technology calibration or to its placement (for example placements on the trunk could be more reliable), but our data suggests that such technology would need to be verified by EEG recordings before it could be reliably used independently in assessment of wake/sleep cycles. It is unlikely that these limitations would apply to rodent models of HD in the same way, since overt dyskinetic movements such as chorea and tics are generally not seen in HD animals although they may exhibit generalised akinesias, hyperactivity and abnormal gait and postural responses. However, our data also indicates that simultaneous Actiwatch^®^ and ambulatory EEG could be useful in the study of nocturnal involuntary movements.

The sleep diary tended to underestimate the extent of wakeful periods as recorded by the EEG and this was especially obvious in the symptomatic individuals. Discrepancies in diary recordings and other methods of sleep recording, including accelerometers, have been reported previously, although in healthy populations it appears that there is a tendency for more sleep disturbance to be reported by diary than is recorded by accelerometers (Kawada, 2008, Short et al., 2012). The under-reporting of EEG-confirmed wakeful periods in our study suggests that individuals do not fill in the diary during a wakeful period and either fail to complete or fail to remember the wakeful period the following morning. However, there were trends towards worse night-time sleep and increased day-time napping in HD positive individuals, which may have reached significance on a group basis with larger numbers. Thus, sleep diaries may still have a useful contribution in this context but, from our data, do not appear to be reliable on an individual basis.

All groups exhibited the expected circadian changes in salivary cortisol levels with morning levels significantly higher than evening levels, indicating that the daily cycles of all subjects were intact. However, we also saw blunting of the morning level in symptomatic individuals. Previous reports have most commonly found cortisol levels to be raised in HD (Aziz et al., 2009, Saleh et al., 2009), although this was not replicated in a recent study in which 24 h sampling of asymptomatic and symptomatic HD patients was performed (Kalliolia et al., 2015). Kalliolia et al. suggested that the raised levels in previous studies could be due to the stress of repeated sampling. The blunted levels in this study may be indicative of circadian disruption, but currently remain unexplained and are deserving of further study in a larger cohort of patients. Salivary cortisol has been used in numerous studies to assay cortisol levels and appears to correlate well with serum levels (Gallagher et al., 2006). There were some problems of acceptability in that some individuals found the collection method distasteful, some patients reported difficulty in producing enough saliva to fill the tube to the 1 ml mark, and there were missing samples across the 7 day collection period. However, overall this appears to be a feasible method for collecting community based samples with relatively little associated stress for the study subjects, as has been noted previously in Alzheimer's disease (Hatfield et al., 2004).

In summary, we highlight some of the difficulties in attempting to undertake community-based studies of circadian rhythm and sleep in an HD population. In addition to technical difficulties that included loss of data due to equipment failure, we found poor agreement between Actiwatch^®^ and ambulatory EEG recordings for patients with both asymptomatic and symptomatic HD, which we interpret as most likely to be due to involuntary movement “breakthrough” during sleep. The data presented in our study is not sufficient to suggest the absence of circadian disturbance in HD, and indeed we consider that the accumulating animal and clinical data point towards circadian disturbance being an intrinsic element of this disorder. However, we believe the findings are important to consider when designing studies of circadian activity and sleep in HD and also in other disorders in which involuntary movements are a feature. We could not confirm the usefulness of sleep diaries from our study, but have data to support further assessment of saliva samples for the assessment of cortisol in this condition.

## Figures and Tables

**Table 1:** Experimental patient and control demographics.

Factor	Symptomatic HD patients (*N* = 9)	Asymptomatic HD patients (*N* = 4)	Controls (*N* = 9)
Mean age (range) in years	51.8 (29–64)	44 (29–56)	41.9 (27–56)
Gender M:F	6:3	1:3	3:6
CAG repeat length (range)	44 (41–52)	40 (38–41)	N/A
Age of onset	44.1 years	N/A	N/A
UHDRS total motor score (mean, range)	42.9 (28–72)	0.3 (0–1)	N/A
UHDRS chorea score (mean, range)	11.1 (5–20)	0	N/A
UHDRS total function capacity (mean, range)	7.6 (4–11)	13 (13–13 a)	N/A
BMI	21.6 (range 17.5–31.3)	27.1 (range 20.6–34.5)	

## References

[bib0005] Aziz N.A., Anguelova G.V., Marinus J., Lammers G.J., Roos R.A.C. (2010). Sleep and circadian rhythm alterations correlate with depression and cognitive impairment in Huntington's disease. Parkinsonism Relat Disord.

[bib0010] Aziz N.A., Pijl H., Frölich M., van der Graaf A.W.M., Roelfsema F., Roos R.A.C. (2009). Increased hypothalamic-pituitary-adrenal axis activity in Huntington's disease. J Clin Endocrinol Metab.

[bib0015] Bode F.J., Stephan M., Wiehager S., Nguyen H.P., Björkqvist M., von Hörsten S. (2009). Increased numbers of motor activity peaks during light cycle are associated with reductions in adrenergic alpha(2)-receptor levels in a transgenic Huntington's disease rat model. Behav Brain Res.

[bib0020] Estrada Sánchez A.M., Mejía-Toiber J., Massieu L. (2008). Excitotoxic neuronal death and the pathogenesis of Huntington's disease. Arch Med Res.

[bib0025] Fish D.R., Sawyers D., Allen P.J., Blackie J.D., Lees A.J., Marsden C.D. (1991). The effect of sleep on the dyskinetic movements of Parkinson's disease, Gilles de la Tourette syndrome, Huntington's disease, and torsion dystonia. Arch Neurol.

[bib0030] Gallagher P., Leitch M.M., Massey A.E., McAllister-Williams R.H., Young A.H. (2006). Assessing cortisol and dehydroepiandrosterone (DHEA) in saliva: effects of collection method. J Psychopharmacol.

[bib0035] Hatfield C.F., Herbert J., van Someren E.J.W., Hodges J.R., Hastings M.H. (2004). Disrupted daily activity/rest cycles in relation to daily cortisol rhythms of home-dwelling patients with early Alzheimer's dementia. Brain.

[bib0040] Hult S., Schultz K., Soylu R., Petersén A. (2010). Hypothalamic and neuroendocrine changes in Huntington's disease. Curr Drug Targets.

[bib0045] Hurelbrink C.B., Lewis S.J.G., Barker R.A. (2005). The use of the Actiwatch-Neurologica system to objectively assess the involuntary movements and sleep-wake activity in patients with mild-moderate Huntington's disease. J Neurol.

[bib0050] Kalliolia E., Silajdžić E., Nambron R., Hill N.R., Doshi A., Frost C. (2014). Plasma melatonin is reduced in Huntington's disease. Mov Disord.

[bib0055] Kalliolia E., Silajdžić E., Nambron R., SJ C., NG M., NR H. (2015). A 24 hour study of the hypothalamic-pituitary axes in Huntington's disease. Mov Disord.

[bib0060] Kantor S., Szabo L., Varga J., Cuesta M., Morton A.J. (2013). Progressive sleep and electroencephalogram changes in mice carrying the Huntington's disease mutation. Brain.

[bib0065] Kawada T. (2008). Agreement rates for sleep/wake judgments obtained via accelerometer and sleep diary: a comparison. Behav Res Methods.

[bib0070] Lazar A.S., Panin F., Goodman A.O., Lazic S.E., Lazar Z.I., Mason S.L. (2015). Sleep deficits but no metabolic deficits in premanifest Huntington's disease. Ann Neurol.

[bib0075] Lebreton F., Cayzac S., Pietropaolo S., Jeantet Y., Cho Yoon H. (2015). Sleep physiology alterations precede plethoric phenotypic changes in R6/1 Huntington's disease mice. PLoS ONE.

[bib0080] Morton A.J., Wood N.I., Hastings M.H., Hurelbrink C., Barker R.A., Maywood E.S. (2005). Disintegration of the sleep-wake cycle and circadian timing in Huntington's disease. J Neurosci.

[bib0085] Morton A.J. (2013). Circadian and sleep disorder in Huntington's disease. Exp Neurol.

[bib0090] Pallier P.N., Maywood E.S., Zheng Z., Chesham J.E., Inyushkin A.N., Dyball R. (2007). Pharmacological imposition of sleep slows cognitive decline and reverses dysregulation of circadian gene expression in a transgenic mouse model of Huntington's disease. J Neurosci.

[bib0095] Pallier P.N., Morton A.J. (2009). Management of sleep/wake cycles improves cognitive function in a transgenic mouse model of Huntington's disease. Brain Res.

[bib0100] Raggi A., Bella R., Pennisi G., Neri W., Ferri R. (2013). Sleep disorders in Parkinson's disease: a narrative review of the literature. Rev Neurosci.

[bib0105] Saleh N., Moutereau S., Durr A., Krystkowiak P., Azulay J.-P., Tranchant C. (2009). Neuroendocrine disturbances in Huntington's disease. PLoS ONE.

[bib0110] Short M.A., Gradisar M., Lack L.C., Wright H., Carskadon M.A. (2012). The discrepancy between actigraphic and sleep diary measures of sleep in adolescents. Sleep Med.

[bib0115] Slats D., Claassen F, Verbeek M.M., Overeem S. (2013). Reciprocal interactions between sleep, circadian rhythms and Alzheimer's disease: focus on the role of hypocretin and melatonin. Ageing Res Rev.

[bib0125] Van Wamelen D.J., Aziz N.A., Anink J.J., van Steenhoven R., Angeloni D., Fraschini F. (2013). Suprachiasmatic nucleus neuropeptide expression in patients with Huntington's Disease. Sleep.

[bib0120] Walker F.O. (2007). Huntington's disease. Lancet.

